# Dual-Axis Pattern of Peripheral Immunity in Myasthenia Gravis Susceptibility: A Prospective Cohort Study and Mendelian Randomization Analysis

**DOI:** 10.3390/biomedicines14102297

**Published:** 2026-10-09

**Authors:** Jiang Lei, Hao Huang, Jiaxin Chen, Xin Huang, Huiyu Feng

**Affiliations:** 1 Department of Neurology, The First Affiliated Hospital of Sun Yat-sen University, Sun Yat-sen University, No. 58 Zhongshan Road, Yuexiu District, Guangzhou 510080, China; 2 Guangdong Provincial Key Laboratory for Diagnosis and Treatment of Major Neurological Disease, No. 58 Zhongshan Road, Yuexiu District, Guangzhou 510080, China; 3 Guangxi Hospital Division of the First Affiliated Hospital, Sun Yat-sen University, No. 3, Foziling Road, Qingxiu District, Nanning 530022, China

**Keywords:** myasthenia gravis, immune cell, peripheral immunity, Mendelian randomization, UK Biobank

## Abstract

**Background:** Previous studies have revealed peripheral immune abnormalities in myasthenia gravis (MG). However, it remains uncertain whether these abnormalities precede the onset of clinical symptoms and affect susceptibility to MG. **Methods:** A prospective study was performed involving 420,936 UK Biobank participants without MG at baseline. Cox proportional hazards models were utilized to evaluate the association between peripheral immune markers and incident MG. Additionally, a two-sample MR analysis was conducted using 731 immune cell traits as exposures and summary statistics for MG from FinnGen as the outcome to assess potential causal effects on MG risk. **Results:** The prospective analysis indicated that elevated neutrophil counts and inflammation-related markers were associated with an increased risk of MG, while higher lymphocyte and monocyte counts were associated with a reduced risk. We identified 22 immune cell traits that showed suggestive associations with MG risk. Among these, dendritic cell/plasmacytoid dendritic cell phenotypes and a higher proportion of CD39^+^ resting CD4^+^ regulatory T cells were associated with lower MG risk, whereas inflammatory monocyte and HLA-DR^+^ activated natural killer cell phenotypes were associated with increased risk. Notably, the proportion of CD14^−^CD16^+^ monocytes showed the strongest risk effect (OR = 2.231, 95% CI 1.370–3.632). After Benjamini–Hochberg FDR correction, none of the 22 associations remained significant, and none showed evidence of colocalization with MG (all PP.H4 < 0.8). **Conclusions:** Peripheral immune alterations were associated with subsequent risk of recorded MG, and exploratory MR analysis suggested a possible dual-axis pattern of pro-inflammatory innate immune activation and impaired immune regulation in MG. These findings require validation in clinically well-characterized MG cohorts.

## 1. Introduction

Myasthenia gravis (MG) is a chronic autoimmune disorder characterized by the presence of autoantibodies that specifically target the neuromuscular junction [1,2]. Its pathogenesis has traditionally been attributed to immune dysregulation, particularly the aberrant activation and impaired regulation of adaptive immunity involving T and B lymphocytes [3,4,5]. However, growing evidence suggests that innate immune cells, including monocytes, dendritic cells (DCs), and natural killer (NK) cells, also contribute to the initiation and persistence of MG [6,7,8]. These findings support the view that MG may involve a dual-axis immune imbalance, characterized by pro-inflammatory innate immune activation alongside impaired immune regulation and tolerance.

Peripheral blood leukocyte differential counts provide a convenient and effective method of assessing peripheral immune function. In addition, C-reactive protein (CRP), the neutrophil-to-lymphocyte ratio (NLR), the platelet-to-lymphocyte ratio (PLR), the lymphocyte-to-monocyte ratio (LMR), and the systemic immune-inflammation index (SII) have been proposed as composite indicators reflecting systemic inflammation and the balance between innate and adaptive immunity [9,10]. However, it is unclear whether peripheral immune dysfunction is a consequence of MG or a risk factor. Numerous observational studies have compared peripheral blood parameters between patients with MG and healthy controls [8,11]. These studies have also examined variations in immune cell counts, proportions, and surface marker expression across different MG subtypes [12,13]. Recent studies have demonstrated an association between CRP and severe fatigue in patients with MG and implicated platelet activation in the pro-inflammatory processes associated with the disease [14,15]. Collectively, these studies suggest associations between multiple immune cell phenotypes and MG. Nonetheless, observational studies are susceptible to confounding by factors such as age, sex, and medication use, as well as reverse causality. These factors complicate the ability to definitively establish a causal relationship between immune cell features and MG. 

Mendelian randomization (MR) is an analytical approach that leverages genetic variants as instrumental variables to infer causal relationships between exposures and outcomes, and it has been extensively utilized in epidemiological research [16,17]. To elucidate the role of peripheral immunity in MG, we first used the UK Biobank (UKB)—a large longitudinal cohort comprising over 500,000 participants—to assess the relationship between peripheral immune markers and incident MG. We then conducted a two-sample MR study to investigate the potential causal associations of 731 immune cells traits on MG risk. This integrated approach was designed to identify peripheral immune alterations associated with subsequent risk of recorded MG and to clarify their roles in MG susceptibility. 

## 2. Methods

### 2.1. Participants

Between 2006 and 2010, the UK Biobank (UKB) recruited over 500,000 individuals aged 40–69. The study design and data collection procedures have been described in detail in the literature [18]. During participants’ baseline assessments, the UKB collected demographic information, lifestyle factors, anthropometric measurements and blood test results, and obtained follow-up outcome data through linkage with the UK National Health Service data system. This study used de-identified UK Biobank data under an approved UK Biobank research application (application number 1196491). The study was conducted in accordance with the Declaration of Helsinki. The UK Biobank has ethical approval from the relevant research ethics committee, and all participants provided informed consent.

**Figure 1:**
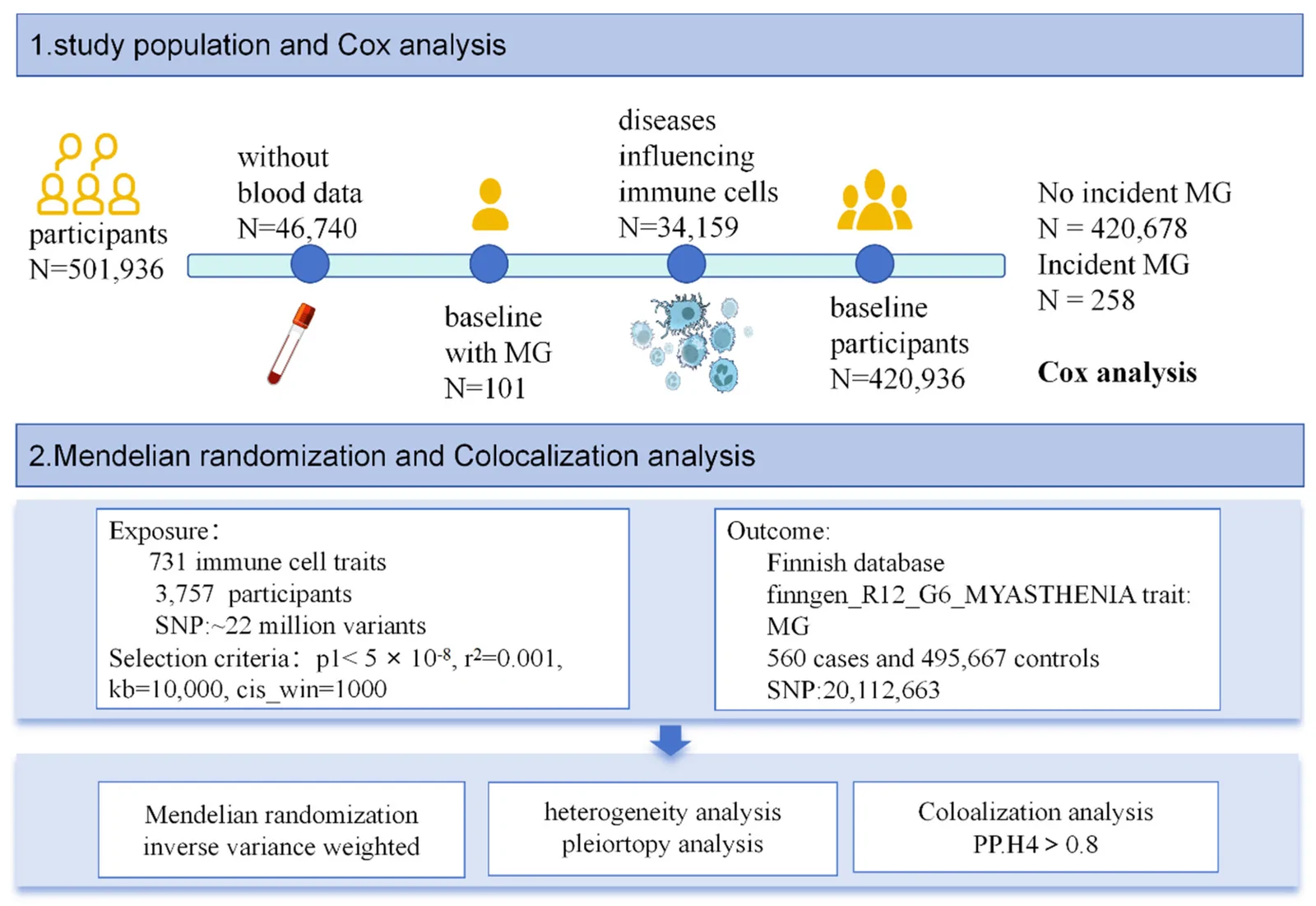
Flowchart of this study. MG Myasthenia gravis, SNP single nucleotide polymorphism.

This study included participants who had completed full blood count and CRP testing at baseline, and who had medical records available for follow-up of outcomes. Participants were excluded hierarchically in three sequential steps. First, we excluded participants with missing data for any of the five peripheral immune markers. Second, we excluded those with a recorded MG diagnosis at or before the baseline assessment date, defined by ICD-10 code G70.0. Third, we excluded those with a baseline record of malignancies, hematological and hematopoietic diseases, other autoimmune diseases, or chronic inflammatory diseases, because these conditions could substantially affect peripheral blood cell counts and CRP levels. The four disease categories in Step 3 were combined into a single exclusion step using a predefined ICD-10-based pattern: malignancies, C00–C99; hematological and hematopoietic diseases, D50–D89; other autoimmune diseases, G35, G36.0, G61.8, G61.0, M05, M06, M32, M35.0, E05.0, and E06.3; and chronic inflammatory diseases, K50–K51. Therefore, participants meeting more than one category were excluded only once. Exclusion criteria were applied at baseline only; incident conditions during follow-up were not used to censor participants (Figure 1).

### 2.2. Peripheral Immunity Markers

Blood samples were analyzed at the UKB Central Laboratory. Peripheral blood cell counts were measured using a clinically validated, fully automated Coulter LH 750 analyser (Beckman Coulter Inc., Brea, CA, USA). CRP concentrations were determined using a high sensitivity immunoturbidimetric assay on a Beckman Coulter AU5800 instrument (Beckman Coulter Inc., Brea, CA, USA). We extracted baseline data on blood parameters, including neutrophils, monocytes, platelets, lymphocytes and CRP, from the initial assessment visit. Based on the peripheral blood cell counts, we calculated four composite immune-inflammatory markers: NLR, PLR, SII and LMR.

### 2.3. Incident MG

MG was defined as the presence of a record containing the International Classification of Diseases, Tenth Revision diagnostic code G70.0. The MG event date was defined as the date of the first record that met this definition during the follow-up period. If the MG event date occurred after the baseline assessment date, the case was defined as incident MG.

### 2.4. Statistical Analysis

The results for continuous variables were presented as means and standard deviations (SD), and categorical variables as counts and percentages. Multivariable Cox proportional hazards regression models were employed to examine the longitudinal relationship between peripheral immune markers and incident MG. Participants who died, withdrew from the study or did not develop MG by the end of the follow-up period on 31 October 2022 were considered censored. Prior to model fitting, peripheral immune markers were standardized to Z-scores to facilitate comparison of effect sizes across markers (Z = (value − mean)/SD). This ensured that the hazard ratio (HR) represented the effect associated with a one-standard-deviation increase in the peripheral immune marker. In Model 1, we adjusted for age and sex. Model 2 was further adjusted for ethnicity, smoking, alcohol consumption, body mass index (BMI) and socioeconomic status, as measured by the Townsend Deprivation Index. All analyses were performed using R software, version 4.5.1.

### 2.5. MR and Bayesian Colocalization Analysis

#### 2.5.1. MR Study Design

This MR analysis was based on three fundamental assumptions: (1) the genetic instrumental variables (single nucleotide polymorphisms, SNPs), was strongly associated with the exposure, namely immune cell traits; (2) these SNPs were independent of any known confounding variables; and (3) the influence of the SNPs on the outcome, MG, was entirely mediated through the exposure, thereby excluding horizontal pleiotropy. Subsequently, colocalization analysis was conducted to ascertain whether the immune cell traits and associated with MG in the MR analysis shared the same causal genetic variants. All data used in this study were obtained from publicly available summary-level data from published GWAS; therefore, no additional ethical approval was required. The workflow of the MR analysis is depicted in Figure 1. 

#### 2.5.2. Data Sources

The data on immune cell traits were derived from an immunophenotyping GWAS involving 3757 individuals of European ancestry [19]. This investigation utilized flow cytometry to assess 731 peripheral blood immune cell traits, which were categorized into four distinct groups: absolute cell counts (*n* = 118), relative cell counts (*n* = 192), median fluorescence intensities (*n* = 389), and morphological parameters (*n* = 32). These traits encompassed major immune cell lineages, including T cells, B cells, natural killer cells, monocytes, dendritic cells, myeloid cells, and regulatory T cells, with GWAS identifiers ranging from ebi-a-GCST90001391 to ebi-a-GCST90002121. Additionally, data pertaining to MG was sourced from the FinnGen Research Project. This dataset included 560 patients with MG, diagnosed based on hospital registry data and International Classification of Diseases codes, and 496,227 controls of European ancestry (https://www.finngen.fi/en, accessed on 18 October 2025).

#### 2.5.3. Instrumental Variable Selection

The exposure variable under investigation is immune cell traits. The criteria for instrumental variable selection were illustrated in Figure 1 and include the following: (1) a significant association with the exposure (*p* < 5 × 10^−8^); (2) independence from other genetic loci, characterized by r^2^ < 0.001 and kb = 10,000; (3) selection of SNPs with F-statistics greater than 10; and (4) exclusion of potential pleiotropic SNPs identified using the PhenoScanner v2 database [20,21]. 

#### 2.5.4. MR Analysis

The primary method employed for MR analysis was inverse variance weighting (IVW), complemented by the MR-Egger mode, weighted mode, and weighted median. Causal effect sizes were reported as odds ratios (OR) with 95% confidence intervals (CI), statistical significance was defined as *p* < 0.05 [22]. Heterogeneity was evaluated using Cochran’s Q statistics under the IVW and MR-Egger methods, with a *p*-value greater than 0.05 indicating no evidence of heterogeneity. MR-Egger intercept test was used to assess pleiotropy. The results were visualized using leave-one-out plots and scatter plots. All MR analyses were conducted using the “TwoSampleMR” package in R version 4.5.1. 

#### 2.5.5. Bayesian Colocalization Analysis

Bayesian colocalization analysis was employed to identify shared causal genetic variants between an exposure and an outcome, thereby to provide further validation for MR results. This analysis considers five distinct hypotheses: H0, no SNPs within the selected locus are associated with either the exposure or the outcome; H1, SNPs within the selected locus are associated with the exposure only; H2, SNPs within the selected locus are associated with the outcome only; H3, both the exposure and outcome are associated within the locus, but with different causal variants; and H4, both the exposure and outcome are associated within the locus and share the same causal variant. A posterior probability for H4 (PP.H4) > 0.8 was considered evidence of a share causal variant [23,24]. The analysis was conducted using the “coloc” package in R version 4.5.1.

## 3. Results

### 3.1. Population Characteristics

Among the 501,936 participants in the UKB cohort, we first excluded 46,740 participants with missing complete blood count or CRP data. We then excluded a further 101 who had already been diagnosed with MG at baseline. To minimize the impact of underlying conditions that could significantly affect peripheral blood cell counts, we further excluded 34,159 participants. Ultimately, 420,936 participants were included in the analysis. During the follow-up period, 258 incident MG cases ware identified. The baseline demographic characteristics of the participants are shown in Table 1. Compared with participants without incident MG, those who developed incident MG were older and had higher BMI. Regarding peripheral immune markers, the Incident MG group had higher neutrophil counts, CRP levels, NLR, PLR and SII than the No incident MG group. There were no significant differences in monocyte, platelet counts, or LMR. Additionally, lymphocyte counts in the Incident MG group tended to be lower, although the difference was not statistically significant.

### 3.2. Peripheral Immune Markers and Incident MG

**Figure 2:**
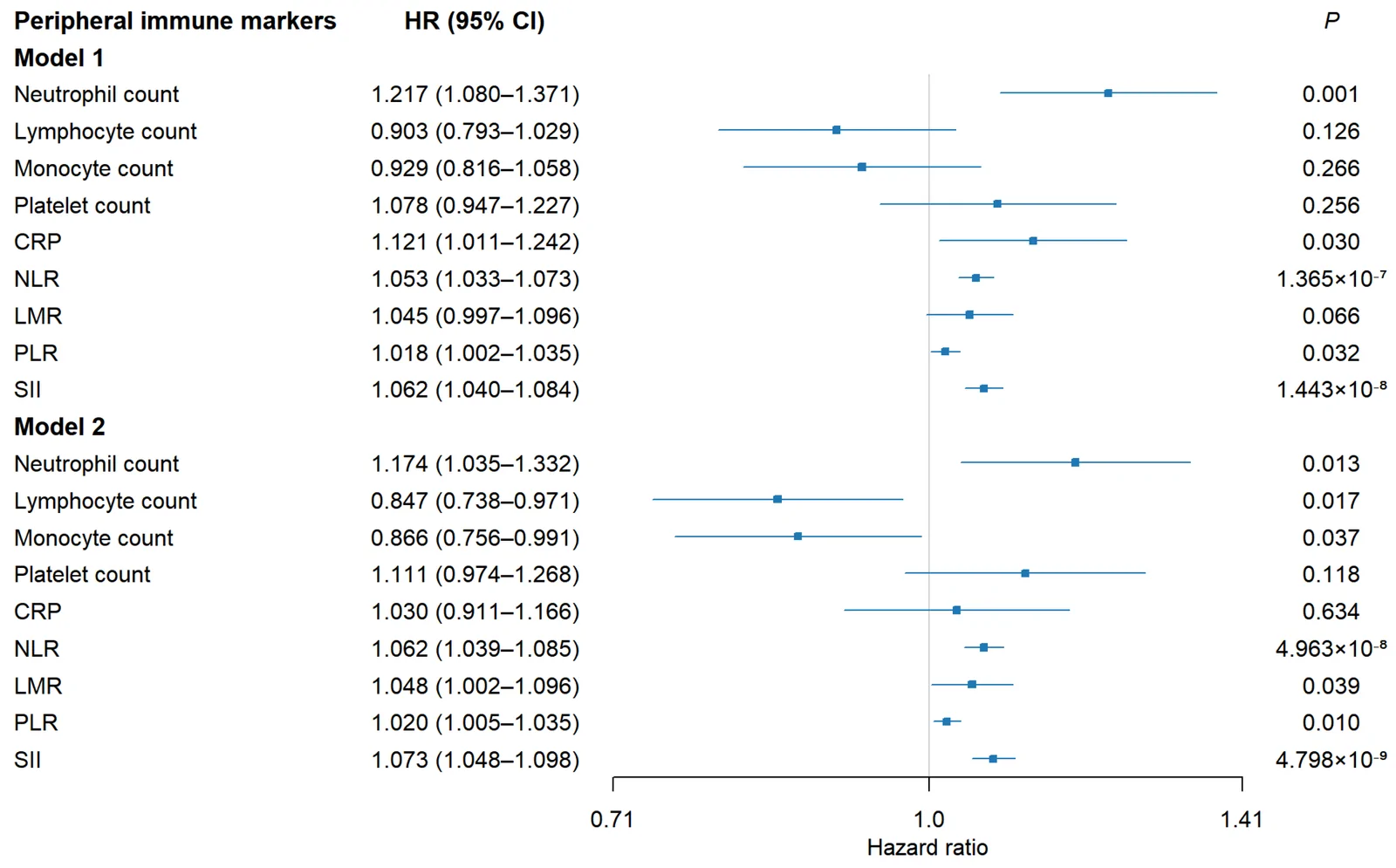
Risk for incident MG according to peripheral immune markers in the UKB. Model 1: adjusted for age and sex. Model 2 was further adjusted for ethnicity, smoking, drinking, BMI and Townsend Deprivation Index. MG Myasthenia gravis, HR = Hazard Ratio, CI = Confidence Interval, BMI body mass index, CRP C-reactive protein, NLR neutrophils/lymphocytes ratio, LMR lymphocytes/monocytes ratio, PLR platelets/lymphocytes ratio, SII systemic immune-inflammation index (neutrophils × platelets/lymphocytes).

Cox proportional hazards regression analysis was used to assess the longitudinal association between peripheral blood immune markers and the risk of incident MG (Figure 2). Model 1, which was adjusted only for age and sex, showed that neutrophil count was associated with an increased risk of MG (HR= 1.217, 95% CI 1.080–1.371, *p* = 0.001). NLR (HR = 1.053, 95% CI 1.033–1.073, *p* = 1.36 × 10^−7^), SII (HR = 1.062, 95% CI 1.040–1.084, *p* = 1.44 × 10^−8^) and PLR (HR = 1.018, 95% CI 1.002–1.035, *p* = 0.032) were all positively associated with MG in Model 1, as was CRP (HR = 1.121, 95% CI 1.011–1.242, *p* = 0.030).

In Model 2, which was further adjusted for ethnicity, smoking, alcohol consumption, BMI and the Townsend deprivation index, the main results remained consistent: Neutrophil count (HR = 1.174, 95% CI 1.035–1.332, *p* = 0.013),NLR (HR = 1.062, 95% CI 1.039–1.085, *p* = 4.96 × 10^−8^), SII (HR = 1.073, 95% CI 1.048–1.098, *p* = 4.80 × 10^−9^), and PLR (HR = 1.020, 95% CI 1.005–1.035, *p* = 0.013) were associated with an increased risk of MG (Figure 2). Furthermore, in Model 2, a higher lymphocyte count was associated with an reduced risk of MG (HR = 0.847, 95% CI 0.738–0.971, *p* = 0.017), as was a lower monocyte count (HR = 0.866, 95% CI 0.756–0.991, *p* = 0.037). Conversely, a higher LMR was associated with an increased risk (HR = 1.048, 95% CI 1.002–1.096, *p* = 0.039). Platelet count and CRP did not reach statistical significance in Model 2.

### 3.3. Instrumental Variables

SNPs were selected according to predefined criteria that ensured a strong correlation with immune cell traits. This rigorous selection process resulted in the identification of 2825 independent instrumental SNPs (Supplementary Table S1). These SNPs demonstrated genome-wide significance in association with their respective immune cell traits and exhibited minimal linkage disequilibrium among themselves. Furthermore, all SNPs had F-statistics exceeding 10, confirming that these instrumental variables effectively reduced the likelihood of weak instrumental bias.

### 3.4. MR Results

**Figure 3:**
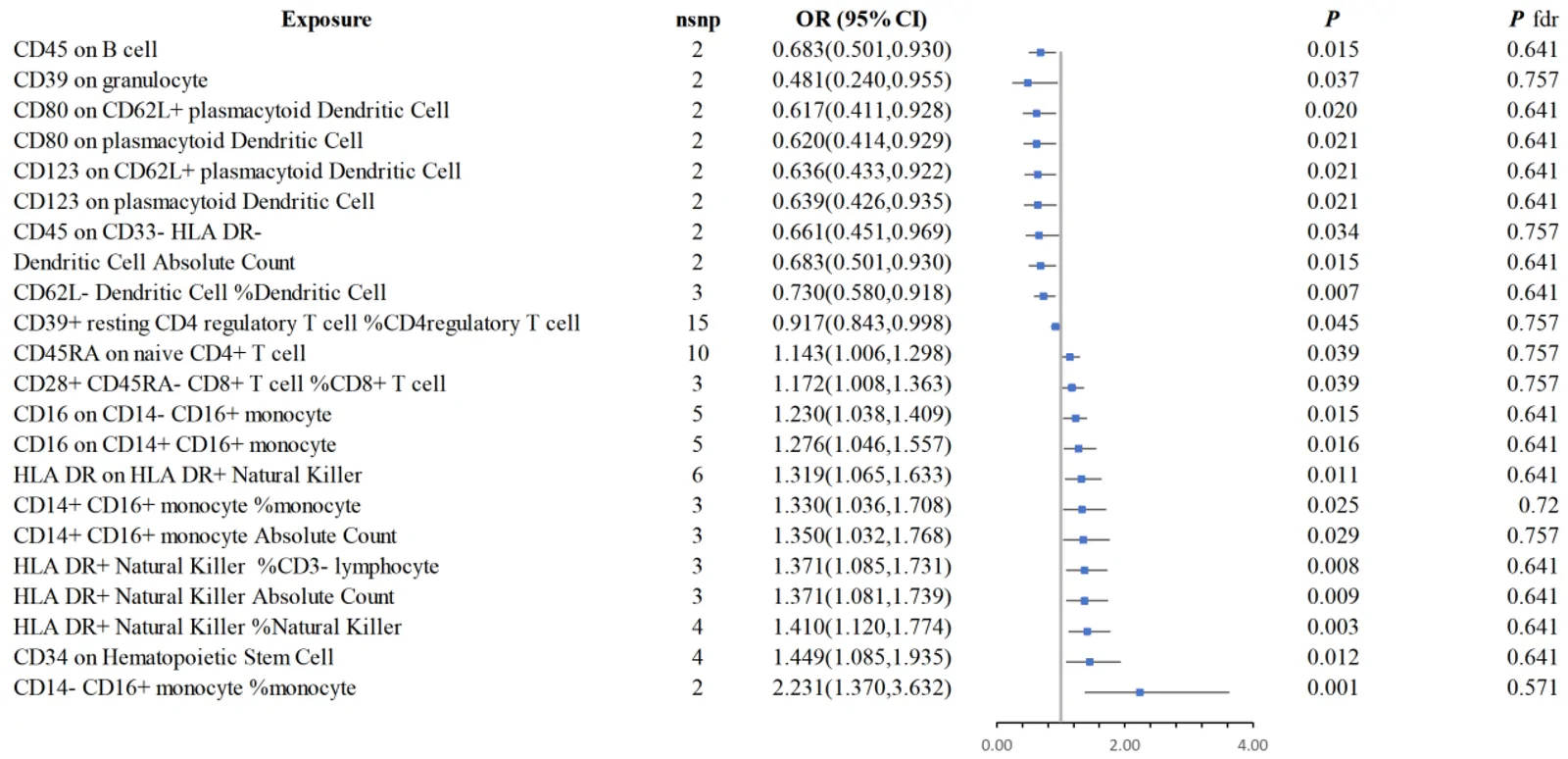
Forest plots showed the results of a MR analysis of the suggestive associations between 22 immune cell traits and MG using IVW. CI confidence interval. OR odds ratios.

Using the IVW method, we found 22 immune cell traits that showed suggestive associations with MG risk (Figure 3). Of these, 10 were associated with reduced risk and 12 with increased risk. Protective associations were mainly observed in traits related to B cells, granulocytes, regulatory T cells, and DC. In particular, CD39 on granulocytes was associated with a markedly reduced risk of MG (OR = 0.481, 95% CI 0.240–0.955, *p* = 0.037), and several DC-related traits also showed consistent protective effects. By contrast, risk-enhancing associations were primarily related to monocyte subsets, NK cells, T-cell subsets, and hematopoietic stem cells. The strongest risk signal was observed for the proportion of CD14^−^CD16^+^ monocytes (OR = 2.231, 95% CI 1.370–3.632, *p* = 0.001). In addition, a higher proportion of CD39^+^ resting CD4 regulatory T cells was associated with lower MG risk, whereas HLA^-^DR^+^ activated NK-cell traits and CD45RA expression on naive CD4^+^ T cells were associated with increased risk. Detailed results from MR Egger, weighted median, and weighted mode analyses are provided in Supplementary Table S2.

**Figure 4:**
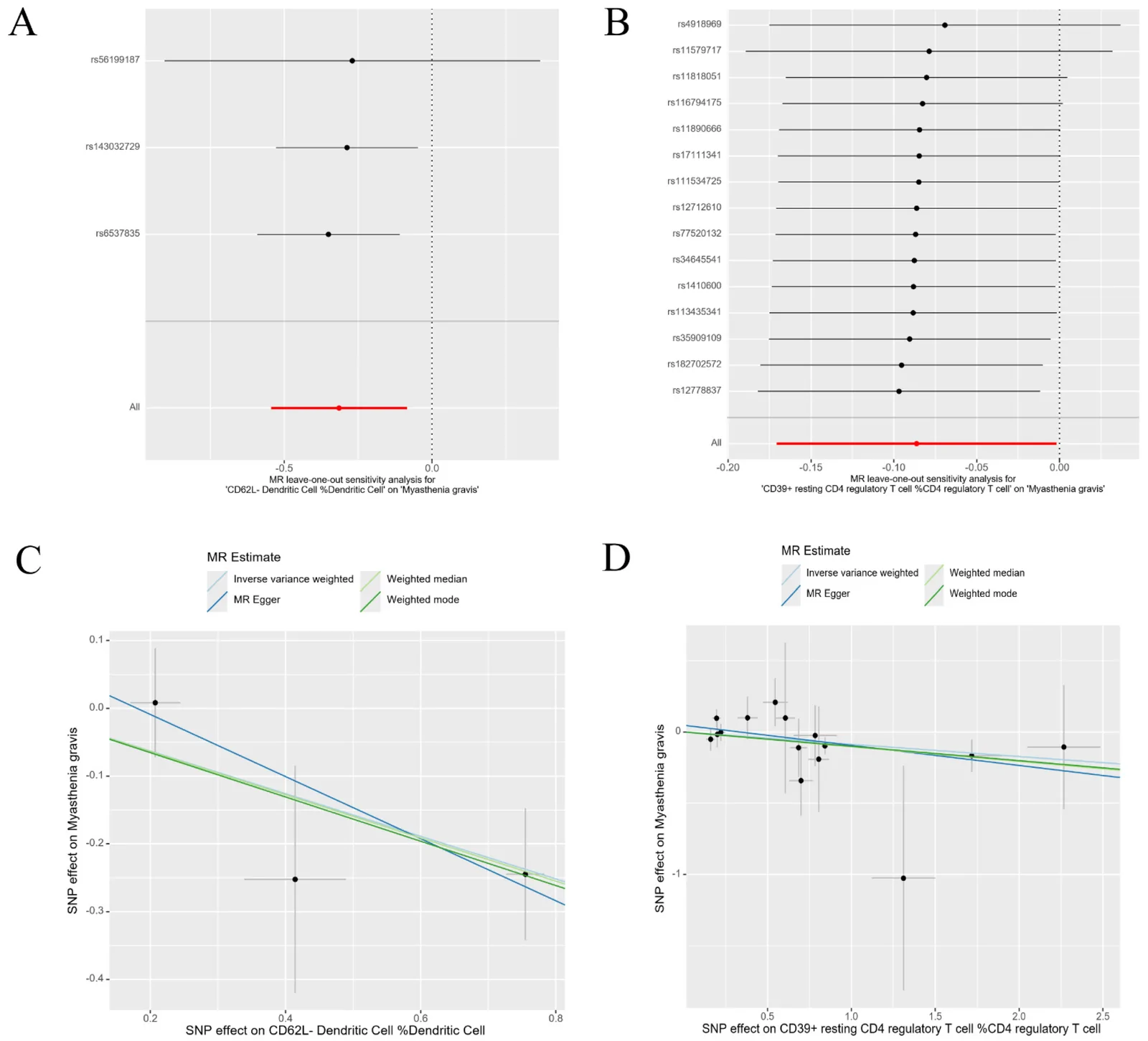
Leave-one-out sensitivity analysis and scatter plots of representative immune cell traits associated with MG. Leave-one-out sensitivity analysis: CD62L^−^ Dendritic Cell %Dendritic Cell on MG (**A**), CD39^+^ resting CD4 regulatory T cell %CD4regulatory T cell on MG (**B**). Scatter plot: CD62L^−^ Dendritic Cell %Dendritic Cell on MG (**C**), CD39^+^ resting CD4 regulatory T cell %CD4regulatory T cell on MG (**D**).

To evaluate the robustness of the MR analysis results, several sensitivity approaches were employed. Cochran’s Q test did not indicate significant heterogeneity under either the MR-Egger or IVW methods for exposures with more than two instrumental variables (all *p* > 0.05; Supplementary Table S3). The MR-Egger intercept was not statistically significant (all *p* > 0.05; Supplementary Table S4). In addition, leave-one-out sensitivity analysis indicated that the primary associations were not influenced by any single SNP. It is noteworthy that the protective factors CD62L^−^DC percentage and CD39^+^ resting CD4 regulatory T cell %CD4 regulatory T cell consistently exhibited robust associations with MG (Figure 4A,B). Additional results from the leave-one-out sensitivity analysis are presented in Supplementary Figure S1. Moreover, scatter plots of instrumental variables against outcomes visually corroborated the MR analysis results. For instance, scatter plots depicting the protective factors CD62L^−^DC percentage and CD39^+^ resting CD4 regulatory T cell %CD4 regulatory T cell in relation to MG are shown in Figure 4C,D. Additional of the scatter plots are presented in Supplementary Figure S2. Finally, we attempted to conduct a reverse MR analysis using MG as an exposure. However, this analysis could not be performed due to the inability to extract instrument variables that met the screening criteria.

Given the large number of immune traits examined (*n* = 731), we applied the Benjamini–Hochberg FDR correction to all MR analyses. After FDR correction, none of the 22 associations remained significant (all adjusted *p* > 0.05) (Figure 3). The MR findings therefore suggested that these 22 immune cell traits showed only suggestive associations with MG.

### 3.5. Colocalization Analysis Results

Bayesian colocalization analysis was performed to determine whether the 22 immune cell traits associated with MG shared causal genetic variants with MG. No evidence of colocalization was observed, as all PP.H4 < 0.8 (Supplementary Table S5).

## 4. Discussion

In this large prospective cohort study combined with an MR analysis, we revealed that elevated neutrophil counts and increased NLR, SII, and PLR were associated with an increased risk of recorded MG, whereas higher lymphocyte and monocyte counts were associated with a reduced risk. Further MR analysis identified 22 immune cell traits showing suggestive associations with MG, including protective associations with regulatory T cell and DC-related phenotypes and risk associations with inflammatory monocytes, activated NK cells and specific T-cell phenotypes. 

Firstly, this prospective population study found that elevated neutrophil counts, as well as elevated NLR, PLR, and SII were all associated with an increased risk of subsequent MG. Previous studies, which were mostly retrospective or cross-sectional analyses, have suggested that patients with MG, particularly during active disease, have higher NLR, PLR, or SII levels; however, it has been difficult to determine whether these are early risk signals or secondary changes resulting from the disease [25,26]. In contrast, this study provides evidence that these abnormalities may be present before recorded MG diagnosis. Furthermore, NLR, PLR, and SII demonstrated more consistent associations than CRP, suggesting that imbalances in immune cell ratios may be a better reflection of MG susceptibility than elevated levels of non-specific inflammation alone. Notably, the observational analysis revealed an inverse association between total monocyte count and risk, while LMR showed a mild positive association. However, MR analysis suggested that the CD14^−^CD16^+^ and CD14^+^CD16^+^ monocyte subsets were associated with an increased risk of MG. The discrepancy between the findings for total monocyte count and subpopulations suggest that the former may mask pathogenic heterogeneity at the latter level, with certain pro-inflammatory or pro-activated monocyte subpopulations potentially being the potentially contributing of MG development.

In this study, we employed MR analysis to further investigate the peripheral immune abnormalities observed in the cohort at the level of specific immune cell subsets and functional phenotypes. Previous studies have paid relatively little attention to DCs, particularly pDCs, in MG; although evidence suggests abnormalities in DC numbers, the relationship between specific subset phenotypes and disease susceptibility remains unclear [27,28]. Our results suggest that the functional state of DCs/pDCs is more important than numerical changes alone. Although CD80 is generally considered a costimulatory molecule, it can also mediate inhibitory signals by binding to CTLA-4 [29,30]. Furthermore, CD39^+^ resting CD4^+^ regulatory T cells are associated with a lower risk of MG. Previous studies have already indicated abnormalities in Treg numbers and function in MG patients [31,32]. CD39 is a key functional molecule in regulatory T cells suppressing inflammatory responses and maintaining immune tolerance by converting extracellular ATP to adenosine [33,34]. Taken together, these findings imply that pDC-associated phenotypes that promote peripheral tolerance, alongside intact CD39-mediated immunosuppressive function, could have a protective effect in the development of MG. However, further functional studies are required to confirm this.

In contrast to the protective factors mentioned above, MR also identified inflammatory monocytes and HLA^−^DR^+^ activated NK cells as being associated with an increased risk of MG. CD16^+^ monocytes typically exhibit stronger characteristics relating to cytokine secretion, tissue migration and antigen presentation [35]. They are also considered to be associated with pro-inflammatory responses and disease activity in various inflammatory diseases [36]. Activated NK cells may amplify innate immune responses by secreting pro-inflammatory cytokines, such as IFN-γ. This cytokine has been shown to promote pathogenic anti- acetylcholine receptor immune responses in experimental autoimmune MG [37,38]. Furthermore, our MR analysis also suggested that higher expression of CD45RA on naïve CD4^+^ T cells is also associated with a higher risk of MG, suggesting that abnormalities in the initial differentiation state of T-cell may contribute to disease susceptibility [39]. In conclusion, these findings raise the possibility that activation of the innate immune system and T-cell dysregulation may work together to drive the development of MG.

Overall, this study has three main strengths. First, the prospective design identifies immune signals associated with subsequent risk of recorded MG. Second, MR analysis explores conventional inflammatory markers at the level of more granular immune subsets. Third, the cohort and MR analyses capture complementary layers of peripheral immune biology: the cohort analysis uses nonspecific markers that reflect systemic inflammation but cannot resolve specific subsets, whereas MR uses genetically predicted immune traits to generate hypotheses about more granular innate and adaptive phenotypes. Together, these two layers of evidence generate the hypothesis that MG susceptibility may involve a possible dual-axis imbalance between pro-inflammatory innate immune activation and impaired immune regulation. This hypothesis requires further validation. 

However, this study also has several limitations. First, although the prospective design strengthens the temporal sequence, observational analysis cannot completely rule out residual confounding or reverse causation. Second, neutrophil counts, CRP, and composite inflammatory indices are highly nonspecific indicators of systemic inflammation, and their modest effect sizes limit their ability to reliably inform individual disease susceptibility. Third, both datasets predominantly included European-ancestry participants, and the UK Biobank is subject to healthy-volunteer bias, limiting generalizability to other ethnic groups. Our findings also reflect population-level susceptibility to recorded MG rather than risk in patients with pre-existing autoimmune diseases. Fourth, MG was defined solely by ICD-10 code G70.0, which has not been independently validated, so outcome misclassification is possible and the conclusions have been restricted to recorded MG cases; with only 258 incident cases, we cannot draw conclusions regarding MG subtypes or severity phenotypes. Finally, the small number of incident cases limits statistical power, none of the MR associations survived FDR correction, and no colocalization was observed; these factors limit causal inference, and the MR results should therefore be regarded as exploratory rather than definitive.

## 5. Conclusions

In summary, this study suggests that peripheral immune alterations were associated with subsequent risk of recorded MG, and exploratory MR analysis suggested a possible dual-axis pattern of pro-inflammatory innate immune activation and impaired immune regulation in MG. These findings require validation in clinically well-characterized MG cohorts.

## Figures and Tables

**Table 1:** Baseline characteristics of UKB participants.

Characteristics	Overall	No Incident MG	Incident MG	*p*
	*n* = 420,936	*n* = 420,678	*n* = 258	
age	56.25 (8.11)	56.25 (8.11)	60.15 (6.79)	<0.001
sex				0.001
Female	235,073 (56%)	234,955 (56%)	118 (46%)	
Male	185,863 (44%)	185,723 (44%)	140 (54%)	
ethnicity				0.013
Non-white	17,147 (4.1%)	17,145 (4.2%)	2 (0.8%)	
White	396,118 (96%)	395,870 (96%)	248 (99%)	
education				0.638
High	184,044 (53%)	183,935 (53%)	109 (51%)	
Low	161,507 (47%)	161,404 (47%)	103 (49%)	
smoking	249,112 (59%)	248,956 (59%)	156 (61%)	0.621
alcohol consumption				0.540
Current	386,208 (92%)	385,977 (92%)	231 (91%)	
Never	18,683 (4.4%)	18,668 (4.4%)	15 (5.9%)	
Previous	15,010 (3.6%)	15,001 (3.6%)	9 (3.5%)	
BMI	27.28 (4.55)	27.28 (4.55)	29.05 (5.12)	<0.001
Townsend	−1.31 (3.08)	−1.31 (3.08)	−1.53 (3.11)	0.245
Neutrophils	4.19 (1.32)	4.19 (1.32)	4.46 (1.41)	0.002
Lymphocytes	1.94 (0.58)	1.94 (0.58)	1.87 (0.59)	0.051
Monocytes	0.47 (0.16)	0.47 (0.16)	0.47 (0.17)	0.733
Platelets	252 (56)	252 (56)	252 (58)	0.841
CRP	2.37 (3.18)	2.37 (3.18)	2.84 (3.42)	0.031
NLR	2.33 (1.15)	2.33 (1.15)	2.80 (2.18)	<0.001
LMR	4.61 (2.88)	4.61 (2.88)	4.69 (3.75)	0.732
PLR	141.51 (67.93)	141.50 (67.91)	154.01 (97.18)	0.040
SII	588.01 (325.71)	587.94 (325.42)	709.83 (627.58)	0.002

Data are presented as the mean (SD) for continuous variables and as a percentage for categorical variables. Higher education refers to college/university/other professional qualification. MG Myasthenia gravis, BMI body mass index, CRP C-reactive protein, NLR neutrophils/lymphocytes ratio, LMR lymphocytes/monocytes ratio, PLR platelets/lymphocytes ratio, SII systemic immune-inflammation index (neutrophils × platelets/lymphocytes).

## Data Availability

The data from the UK Biobank that has been utilised in this study can be accessed upon application and subsequent approval from the UK Biobank (https://ams.ukbiobank.ac.uk/ams/, accessed on 18 October 2025). The summary statistics for myasthenia gravis were obtained from the FinnGen Research Project (https://www.finngen.fi/en, accessed on 18 October 2025). The immune cell trait GWAS summary data were obtained from studies that had been published in the public domain. Should further information supporting the findings of this study be required, the corresponding author will be available to provide it upon request, in accordance with the data access policies of the original data providers.

## Supplementary Materials

The following supporting information can be downloaded at: https://www.mdpi.com/article/10.3390/biomedicines14102297/s1, Table S1: SNPs screened according to methodological criteria; Table S2: The MR Egger, weighted median, and weighted mode analysis results; Table S3: Heterogeneity tests; Table S4: Pleiotropy tests; Table S5: Co-localization between 22 immune cell traits and MG; Figure S1: Leave-one-out sensitivity analysis; Figure S2: Scatter plot.

## Abbreviations

BMI	body mass index
CI	confidence interval
CRP	C-reactive protein
DC	dendritic cell
HR	hazard ratio
IVW	inverse-variance weighted
LMR	lymphocyte-to-monocyte ratio
MG	myasthenia gravis
MR	Mendelian randomization
NK cells	natural killer cells
NLR	neutrophil-to-lymphocyte ratio
OR	odds ratio
pDCs	plasmacytoid dendritic cells
PLR	platelet-to-lymphocyte ratio
PP.H4	posterior probability of hypothesis 4
SD	standard deviation
SII	systemic immune-inflammation index
SNPs	single nucleotide polymorphisms
UKB	UK Biobank
