# Supplementary material for: Dual-Axis Pattern of Peripheral Immunity in Myasthenia Gravis Susceptibility: A Prospective Cohort Study and Mendelian Randomization Analysis
Source: Biomedicines. 2026 Oct 9;14(10):2297. doi: 10.3390/biomedicines14102297 (PMC13701481; doi:10.3390/biomedicines14102297)
Supplement: Supplementary file 1 [file biomedicines-14-02297-s001.zip › Supplementary Figures S1 and S2.pdf]

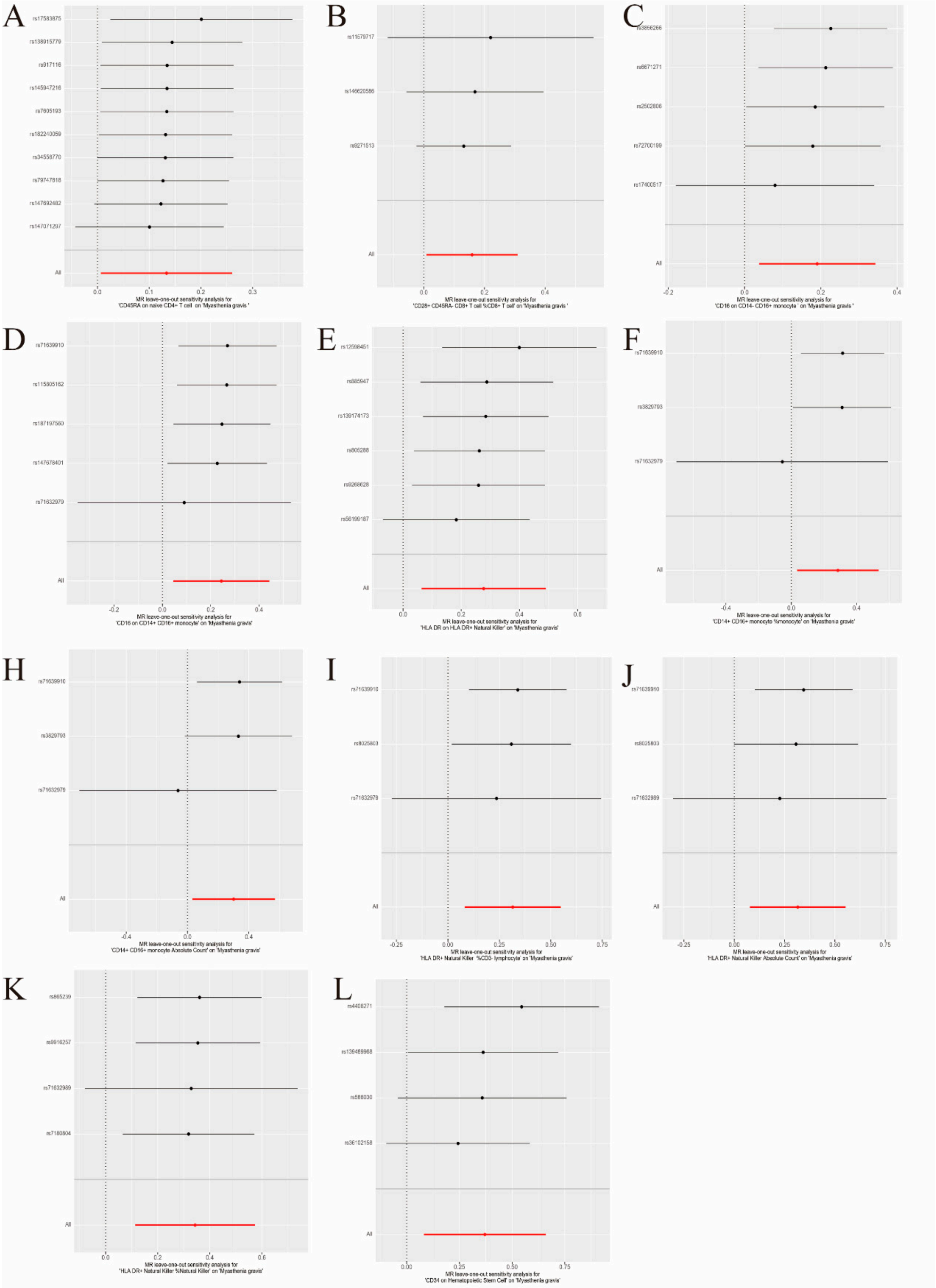

Supplementary Figure S1. Leave-one-out sensitivity analysis. CD45RA on naive $CD4^{+}$ T cell on MG (A). $CD28^{+}$ $CD45RA^{-}$ $CD8^{+}$ T cell %$CD8^{+}$ T cell on MG(B). CD16 on $CD14^{-}$ $CD16^{+}$ monocyte on MG (C).CD16 on $CD14^{+}$ CD16+ monocyte on MG(D). HLA DR on HLA DR+ Natural Killer on MG (E). $CD14^{+}$ $CD16^{+}$ monocyte %monocyte on MG (F). $CD14^{+}$ $CD16^{+}$ monocyte Absolute Count on MG (G). HLA DR^{+} Natural Killer %CD3- lymphocyte on MG (H). HLA DR+ Natural Killer Absolute Count on MG (I). HLA DR^{+} Natural Killer %Natural Killer on MG (J). CD34 on Hematopoietic Stem Cell (K).

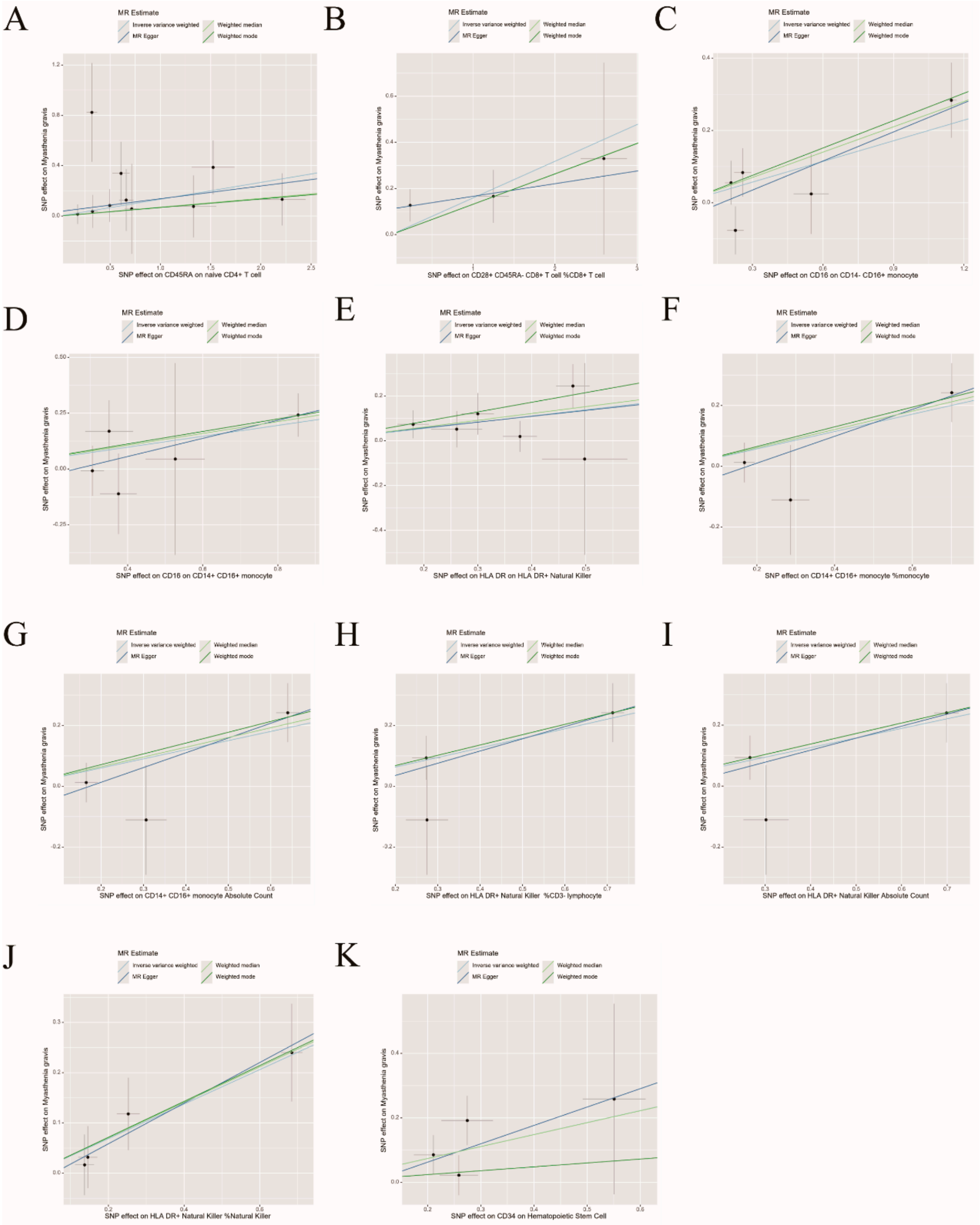

Supplementary Figure S2. Scatter plot: CD45RA on naive CD4$^+$ T cell on MG (A). CD28$^+$ CD45RA$^-$ CD8$^+$ T cell %CD8$^+$ T cell on MG(B). CD16 on CD14$^-$ CD16$^+$ monocyte on MG (C).CD16 on CD14$^+$ CD16$^+$ monocyte on MG(D). HLA DR on HLA DR$^+$ Natural Killer on MG (E). CD14$^+$ CD16$^+$ monocyte %monocyte on MG (F). CD14$^+$ CD16$^+$ monocyte Absolute Count on MG (G). HLA DR$^+$ Natural Killer %CD3$^-$ lymphocyte on MG (H). HLA DR$^+$ Natural Killer Absolute Count on MG (I). HLA DR$^+$ Natural Killer %Natural Killer on MG (J). CD34 on Hematopoietic Stem Cell (K).
